# Facteurs prédictifs de la réponse à la CERA chez les hémodialysés chroniques naïfs de traitement par agent stimulant l’érythropoïèse

**DOI:** 10.11604/pamj.2015.20.331.4467

**Published:** 2015-04-07

**Authors:** Mariam Ezziani, Adil Najdi, Souad Mikou, Hakim Hanin, Mohammed Arrayhani, Tarik Sqalli Houssaini

**Affiliations:** 1 Service de Néphrologie, CHU Hassan II, Fès, Maroc; 2 Laboratoire d'Epidémiologie, de Recherche Clinique et de Sante Communautaire, Faculté de Médecine et de Pharmacie de Fès, Maroc

**Keywords:** Hémodialyse chronique, anémie, hémoglobine, ASE, CERA, chronic hemodialysis, anemia, hemoglobin, ASE, CERA

## Abstract

La correction et la stabilité du taux d'hémoglobine est un objectif majeur du traitement de l'anémie chez les hémodialysés chroniques. Toutefois, la cible d'hémoglobine > 11g/dl fixée par les recommandations demeure difficile à atteindre dans notre contexte. Le but de cette étude est d’évaluer la réponse au traitement par CERA (continuous erythropoietin receptor activator) chez une population d'hémodialysés chroniques naïfs de tout traitement par agent stimulant de l’érythropoïèse et étudier les différents facteurs associés à une mauvaise réponse au traitement. Il s'agit une étude prospective mono centrique faite au sein d'une population d’ hémodialysés chroniques. Ont été inclus les patients en hémodialyse depuis plus de 12 mois, naïfs de tout traitement par agent stimulant de l’érythropoïèse (ASE) et ayant un taux d'hémoglobine(Hb) < 10g/dl. L'administration régulière de la CERA et l'ajustement des doses ont été faits selon les recommandations. L’évaluation de la réponse, en fin de traitement, a porté sur l'atteinte ou non d'un taux d'hémoglobine cible > 11g/dl. Sur 87 patients en hémodialyse périodique, 22 (25,3%) sont naïfs de tout traitement par ASE. Il s'agit de 13 hommes et 9 femmes avec un âge moyen de 46 ± 19 ans et une ancienneté en hémodialyse de 67 ± 59 mois. Le taux initial d'hémoglobine est de 7,8 ± 1,3 g/dl. Au bout de 4 mois de traitement régulier par la CERA, le taux final d'Hb est de 10,9 ± 2,1g/dl et 63,6% des patients ont atteint la cible d'Hb > 11g/dl. La dose moyenne de CERA à la fin de l’étude est de 0,89 ± 0,35 µg/kg/15j. L'analyse des facteurs prédictifs montre que la réponse finale dépend du taux d'Hb initial (p = 0,002). En effet, quand le taux d'Hb initial est > 8 g/dl, le taux de réponse est de 88% vs 46% lorsque le taux d'Hb < 8g/dl (p1-3]. Cette complication commune de la maladie rénale chronique est multifactorielle dont le déficit en érythropoïétine est le principal facteur causal [4]. Dès l'avènement des agents stimulants de l’érythropoïèse (ASE) en 1980 et le développement des recommandations de bonnes pratiques médicales, la gestion de l'anémie rénale est devenue meilleure avec une amélioration de la qualité de vie des patients [5–9]. Les recommandations de bonne pratique suggèrent actuellement le maintien de l'Hb à un taux > 11 g/dl sans dépasser 13 g/dl [9, 10]. Cependant, le maintien d'un taux d'hémoglobine stable nécessite du temps, des ressources humaines et financières et exige des adaptations thérapeutiques fréquentes [11]. Le méthoxy polyéthylène glycol-époétine bêta, activateur continu du récepteur de l’érythropoïétine (C.E.R.A), est un nouvel ASE qui permet la correction de l'anémie et la stabilité du taux d'hémoglobine au rythme d'une injection mensuelle [12]. Le but de cette étude est d’évaluer la réponse au traitement par CERA chez une population d'hémodialysés chroniques naïfs de tout traitement par ASE et d’étudier les différents facteurs associés à la mauvaise réponse au traitement

## Introduction

La prévalence de l'anémie au cours de la maladie rénale chronique et chez les hémodialysés chroniques est très élevée, elle est associée à une morbidité et une mortalité significatives [1–3]. Cette complication commune de la maladie rénale chronique est multifactorielle dont le déficit en érythropoïétine est le principal facteur causal [4]. Dès l'avènement des agents stimulants de l’érythropoïèse (ASE) en 1980 et le développement des recommandations de bonnes pratiques médicales, la gestion de l'anémie rénale est devenue meilleure avec une amélioration de la qualité de vie des patients [5–9]. Les recommandations de bonne pratique suggèrent actuellement le maintien de l'Hb à un taux > 11 g/dl sans dépasser 13 g/dl [9, 10]. Cependant, le maintien d'un taux d'hémoglobine stable nécessite du temps, des ressources humaines et financières et exige des adaptations thérapeutiques fréquentes [11]. Le méthoxy polyéthylène glycol-époétine bêta, activateur continu du récepteur de l’érythropoïétine (C.E.R.A), est un nouvel ASE qui permet la correction de l'anémie et la stabilité du taux d'hémoglobine au rythme d'une injection mensuelle [12]. Le but de cette étude est d’évaluer la réponse au traitement par CERA chez une population d'hémodialysés chroniques naïfs de tout traitement par ASE et d’étudier les différents facteurs associés à la mauvaise réponse au traitement

## Méthodes

C'est une étude prospective mono centrique sur une période de 4 mois conduite entre Avril et Aout 2011. Les patients éligibles sont des adultes (âge > 18 ans), naïfs de tout traitement par ASE, en hémodialyse chronique depuis plus de 1 an et ayant une anémie définie par un taux d'hémoglobine < 10g/dl. Les autres critères d'inclusion et d'exclusion sont énumérés dans le Tableau 1. Chez ces patients, la CERA a été administrée à une dose initiale de 0,6µg/Kg en injection intraveineuse en fin de séance de dialyse tous les 15 jours. L’évaluation de la réponse se fait par la réalisation systématique d'une numération de la formule sanguine (NFS) toutes les 2 semaines. Les adaptations posologiques sont mensuelles; si le taux d'Hb augmente de 1 à 2 g/dl/mois, on maintient la même dose initiale, par contre, si au bout d'un mois, la variation du taux d'Hb est > 2g/dl ou < 1g/dl, la posologie doit être réduite ou augmentée de 25% par rapport à la dose initiale respectivement. Une fois, la cible est atteinte, définie par un taux d'hémoglobine entre 11-13g/dl, la dose mensuelle d'entretien est le double de celle ayant permis d'atteindre cet objectif. A noter que les patients recevaient une supplémentation en fer injectable pour maintenir un statut martial correct. Au terme des 4 mois de traitement par la CERA, le critère principal de jugement est la proportion des patients ayant un taux d'hémoglobine > 11g/dl considérés comme répondeurs. L'analyse statistique a été réalisée par un logiciel SPSS version 17, en utilisant le test Khi-deux et le test exact de Fisher pour la comparaison des variables qualitatives et le test de Student pour la comparaison des variables quantitatives avec un risque d'erreur de 1ère espèce de 5%. Une analyse uni-variée des différents paramètres a permis de déterminer les facteurs de mauvaise réponse au traitement à la CERA.

**Tableau 1:** Les critères d'inclusion et d'exclusion des patients

**Critères d'inclusion**
Patients adultes (âge ≥ 18 ans)
Anémie d'origine rénale définie par un taux d'hémoglobine < 10 g/dl
Durée en hémodialyse chronique >12mois
Un statut martial adéquat avec un taux de ferritinémie >200µg/l
Patients naïfs de tout traitement par agent stimulant l’érythropoïèse
**Critères d'exclusion**
Hospitalisation récente
Syndrome hémorragique (digestif ou autre) avant (2mois) ou au cours de l’étude
Autres causes non rénales d'anémie (hémoglobinopathies, hémolyse…)
Néoplasie active
Maladie inflammatoire chronique symptomatique ou non contrôlée (tuberculose.)
C-réactive protéine > 15 mg/l
Hypertension artérielle non contrôlée (PAS ≥ 160 mmHg et/ou PAD ≥ 100 mmHg)

## Résultats

**Figure 1:**
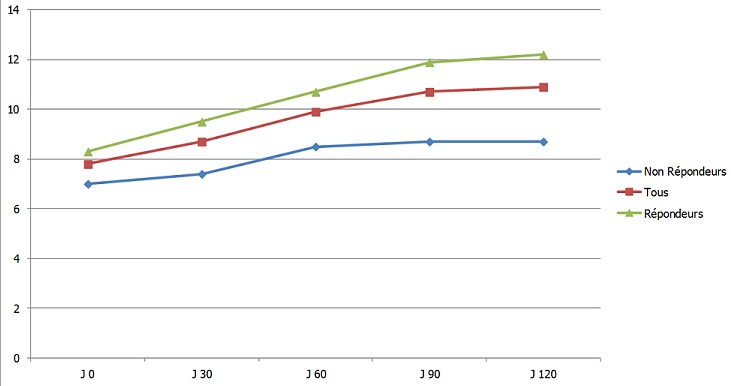
Évolution du taux d'hémoglobine durant l’étude

**Figure 2:**
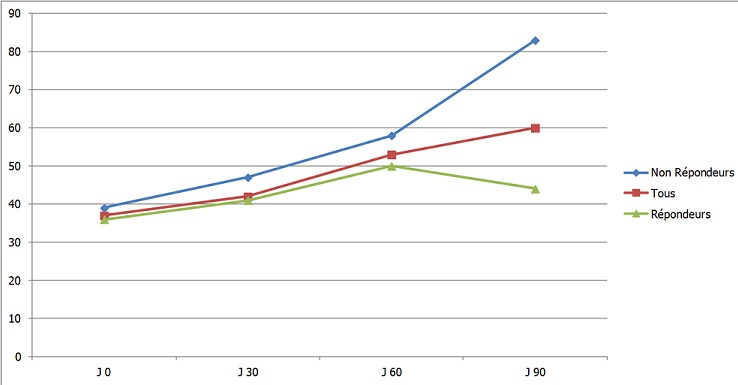
Évolution des doses de CERA en Hg/15j au cours de l’étude

Sur un total de 87 patients hémodialysés dans notre centre, 22 étaient naïfs de tout traitement par ASE (25%), avec un sex ratio de 1,44 (13H/9F), un âge moyen de 46 ± 19 ans et une ancienneté en hémodialyse de 67 ± 59 mois. Le taux initial moyen d'Hb est de 7,8 ± 1,3 g/dl. Le Tableau 2 résume les caractéristiques démographiques et biologiques de la population étudiée. Après quatre mois de traitement par CERA, 14 patients ont atteint la cible d'hémoglobine > 11 g/dl, soit 63,6% avec une médiane de temps de réponse de dix semaines. Le taux final moyen d'hémoglobine est de 10,9 ± 2,1 g/dl et le taux moyen de CERA à la fin de l’étude est de 0,89 ± 0,35 µg/kg/15jours (Tableau 3). Pour les patients répondeurs, l'augmentation du taux d'Hb sur 4 mois était de 3,8 ± 1,3 g/dl passant d'un taux initial de 8,3 ± 1,3 g/dl à 12,2 ± 0,9 g/dl. Pour le groupe des non répondeurs, le taux d'Hb est passé de 7 ± 0,8 à 8,6 ± 1,2 g/dl, soit une élévation de 1,7 ± 1,3 g/dl après 4 mois de traitement. La dose de CERA dans le groupe des répondeurs était de 0,69 ± 0,08 µg/kg/15j contre 0,83 ± 0,22 µg/kg/15jr dans l'autre groupe. Les Figure 1 et Figure 2 montrent l’évolution du taux d'Hb et des doses de CERA au cours de l’étude. L'analyse des caractéristiques cliniques et biologiques des répondeurs et non répondeurs montre qu'il n'y'a pas de différence significative entre les deux groupes en ce qui concerne l’âge, le sexe, l'ancienneté en hémodialyse, la qualité d'hémodialyse, le statut martial, l’état inflammatoire jugé sur le taux de CRP, l’état nutritionnel jugé sur le taux d'albuminémie, le taux de PTH intacte, le statut sérologique de l'hépatite virale C et la dose moyenne de CERA. La seule différence significative est le taux d'hémoglobine initial qui est plus bas dans le deuxième groupe 7 ± 0,8 g/dl contre 8,3 ± 1,3 g/dl avec un p à 0,002 (Tableau 4). Le taux de réponse passe de 46% à 88% quand le taux initial d'Hb est >8 g/dl (p < 0,04) (Tableau 4).

**Tableau 2:** Les caractéristiques démographiques et biologiques des patients

Paramètres	Moyennes ± écart-type
Sex ratio (H/F)	13 / 9
Age (années)	46 ± 19
Ancienneté en hémodialyse (mois)	67 ± 59
Hémoglobine initiale (g/dl)	7,8 ± 1,3
Ferritinémie (µg/l)	403 ± 269
CRP (mg/l)	9,1 ± 2,4
Albumine (mg/l)	41,7 ± 4,9
PTH intacte (pg/ml)	634 ± 737
Heures dialyse/semaine: 12h/10h	13 / 9
VHC +	32%

Abréviations: CRP, c réactive protéine; PTH, parathormone; VHC, virus de l'hépatite C

**Tableau 3:** La réponse après quatre mois de traitement par CERA

Paramètres	Début CERA	Fin CERA
Hémoglobine (g/dl)	7,8 ± 1,3	**10,9 ± 2,1**
[min - max]	[5,8 – 9,9]	[6,5 – 14,4]
Dose moyenne de CERA en µg/kg/15j	0,6	0,89 ± 0,35
Dose moyenne de CERA en µg/kg/15j sur 4 mois	0,75 ± 0,13	
[min – max]	[0,6-1,35]	

**Tableau 4:** Comparaison des caractéristiques des patients répondeurs et non répondeurs

Paramètres	Répondeurs	Non répondeurs	*P*
Sexe H/F (%)	69 / 45	31 / 55	NS
Age (années)	44 ± 20	48 ± 16	NS
Ancienneté en HD (mois)	73 ± 58	55 ± 62	NS
Hémoglobine initiale (g/dl)	**8,3 ± 1,3**	**7 ± 0,8**	**0,02**
Variation du taux d'hémoglobine	**3,8 ± 1,3**	**1,7 ± 1,3**	**< 0,001**
Hémoglobine finale (g/dl)	**12,2 ± 0,9**	**8,6 ± 1,2**	**< 0,001**
Dose CERA (µg/Kg/15j)	0,69 ± 0,08	0,83 ± 0,22	NS
Ferritinémie (µg/l)	391 ± 251	424 ± 315	NS
CRP (mg/l)	9,2 ± 2,1	9,1 ± 2,9	NS
Albumine (g/l)	42,3 ± 3,9	40,9 ± 6,6	NS
PTH intacte (pg/l)	756 ± 816	421 ± 559	NS
Heure dialyse/sem 12h/10h (%)	54 / 78	46 / 22	NS
VHC +	40%	60%	NS

## Discussion

Peu d’études ont inclus des patients hémodialysés et naïfs de tout traitement par ASE pour évaluer l'efficacité de la CERA. Dans la majorité des études les patients sont déjà sous ASE avant le stade d'hémodialyse ou dès la mise en hémodialyse. La particularité de cette étude est que nos patients n'ont jamais reçus de traitement par ASE et ce malgré une ancienneté moyenne en hémodialyse de plus de cinq ans, faute de moyens financiers. Dans l’étude AMICUS, l'efficacité de la CERA administrée chaque deux semaines a été comparée à l’époétine administrée trois fois par semaines chez des patients dialysés naïfs de traitement par ASE dans les trois mois précédents le début de l’étude [13]. Le nombre de patients traités par CERA dans cette étude était de 135 patients. Ces patients sont en moyenne plus âgés que les notre et se comparent aux 22 patients de notre étude en ce qui concerne le sexe, l’état inflammatoire, l’état nutritionnel et le statut martial (Tableau 5). Le taux de réponse était de 93% dans l’étude AMICUS contre 64% dans notre étude. La médiane de réponse dans l’étude AMICUS est de 8 semaines contre 10 semaines pour nos patients. Ces résultats peuvent être expliqués par le taux d'Hb initial de nos patients qui est plus bas 7,8 g/dl contre 9,4 g/dl ainsi que la durée courte d’évaluation de notre étude qui est de 4 mois contre 6 mois. A noter que le taux de réponse dans notre étude passe à 88% quand le taux initial d'Hb est > 8 g/dl. Notre étude a des limites à savoir le faible effectif, la courte durée qui ne permet pas d'atteindre la cible d'hémoglobine étant donné le taux initial bas et ne permet pas aussi d’évaluer la stabilité de l'hémoglobine dans le temps.

**Tableau 5:** Comparaison des résultats de notre étude avec l’étude AMICUS

Paramètres	Notre étude (N = 22)	AMICUS (N = 135)
Sexe masculin (%)	59	65
Age (années)	46	54
Albumine (g/l)	41	39
CRP (mg/l)	9	5
Ferritinémie (µg/l)	403	376
Hémoglobine initiale (g/dl)	**7,8**	**9,4**
Variation du taux d'Hb en phase de correction	3,1	2,7
Hémoglobine finale (g/dl)	10,9	12,1
% de réponse à la CERA (quand Hb > 8g/dl)	64	93
	88	
Médiane temps de réponse (semaines)	10	8

## Conclusion

L'anémie est une complication majeure de la maladie rénale chronique, elle est souvent traitée tardivement ou parfois non traitée par les ASE. Ces derniers sont très efficaces. La CERA offre la possibilité d'une administration bi ou mensuelle. Le traitement précoce de l'anémie est indispensable pour réduire la morbidité et la mortalité qui en résulte.

## References

[1] Collins AJ (2001). Death, hospitalization, and economic associations among incident hemodialysis patients with hematocrit values of 36 to 39%. J Am Soc Nephrol..

[2] Levin A (2001). Prevalence of cardiovascular damage in early renal disease. Nephrol Dial Transplant..

[3] Weiner DE (2004). Chronic kidney disease as a risk factor for cardiovascular disease and all-cause mortality:a pooled analysis of community-based studies. J Am Soc Nephrol..

[4] Panchapakesan U, Siska S, Pollock C (2007). Nanomedicines in the treatment of anemia in renal disease: focus on CERA (Continuous Erythropoietin Receptor Activator). International Journal of Nanomedicine..

[5] Barrett BJ (1999). Clinical practice guidelines for the management of anemia coexistent with chronic renal failure: Canadian Society of Nephrology. J Am Soc Nephrol..

[6] Locatelli F (2004). European Best Practice Guidelines Working Group: Revised European best practice guidelines for the management of anemia in patients with chronic renal failure. Nephrol Dial Transplant..

[7] Pollock C, McMahon L (2005). Biochemical and hematological targets guidelines Haemoglobin. Nephrology..

[8] US Renal Data System (2005). USRDS 2005 Annual Data Report, Bethesda, National Institutes of Health, National Institute of Diabetes and Digestive and Kidney Diseases.

[9] National Kidney Foundation (2006). KDOQI clinical practice guidelines and clinical practice recommendations for anemia in chronic kidney disease. Am J Kidney Dis..

[10] Locatelli F (2004). European Best Practice Guidelines Working Group: Revised European best practice guidelines for the management of anaemia in patients with chronic renal failure. Nephrol Dial Transplant..

[11] De Cock E, Van Bellingham L, Standaert B (2002). Assessing provider time for anemia management of dialysis patients using time & motion methods: a multi-centre observational study in Europe. Value Health..

[12] Macdougall IC (2005). CERA (continuous erythropoietin receptor activator): A new erythropoiesis-stimulating agent for the treatment of anemia. Curr Hematol Rep..

[13] Marian Klinger (2007). Efficacy of Intravenous Methoxy Polyethylene Glycol-Epoetin Beta Administered Every 2 Weeks Compared With Epoetin Administered 3 Times Weekly in Patients Treated by Hemodialysis or Peritoneal Dialysis: A Randomized Trial. American Journal of Kidney Diseases.

